# Impact of type of oral anticoagulants in patients with cerebral microbleeds after atrial fibrillation-related ischemic stroke or TIA: Results of the NOACISP-LONGTERM registry

**DOI:** 10.3389/fneur.2022.964723

**Published:** 2022-09-20

**Authors:** Benjamin Wagner, Lisa Hert, Alexandros A. Polymeris, Sabine Schaedelin, Johanna M. Lieb, David J. Seiffge, Christopher Traenka, Sebastian Thilemann, Joachim Fladt, Valerian L. Altersberger, Annaelle Zietz, Tolga D. Dittrich, Urs Fisch, Henrik Gensicke, Gian Marco De Marchis, Leo H. Bonati, Philippe A. Lyrer, Stefan T. Engelter, Nils Peters

**Affiliations:** ^1^Department of Neurology and Stroke Center, University Hospital Basel and University of Basel, Basel, Switzerland; ^2^Department of Intensive Care Medicine, University Hospital Basel and University of Basel, Basel, Switzerland; ^3^Clinical Trial Unit, University Hospital Basel and University of Basel, Basel, Switzerland; ^4^Department of Diagnostic and Interventional Neuroradiology, Clinic for Radiology and Nuclear Medicine, University Hospital Basel and University of Basel, Basel, Switzerland; ^5^Department of Neurology, Inselspital, Bern University Hospital, University of Bern, Bern, Switzerland; ^6^Neurology and Neurorehabilitation, University Department of Geriatric Medicine Felix Platter and Department of Clinical Research, University of Basel, Basel, Switzerland; ^7^Stroke Center, Klinik Hirslanden Zurich, Zurich, Switzerland

**Keywords:** stroke, atrial fibrillation, anticoagulation, direct-acting oral anticoagulant, cerebral microbleeds, small vessel disease, vitamin K anticoagulants

## Abstract

**Background:**

Cerebral microbleeds (CMBs) may have a differential impact on clinical outcome in stroke patients with atrial fibrillation (AF) treated with different types of oral anticoagulation (OAC).

**Methods:**

Observational single-center study on AF-stroke-patients treated with OAC. Magnetic-resonance-imaging was performed to assess CMBs. Outcome measures consisted of recurrent ischemic stroke (IS), intracranial hemorrhage (ICH), death, and their combined analysis. Functional disability was assessed by mRS. Using adjusted logistic regression and Cox proportional-hazards models, we assessed the association of the presence of CMBs and OAC type (vitamin K antagonists [VKAs] vs. direct oral anticoagulants [DOACs]) with clinical outcome.

**Results:**

Of 310 AF-stroke patients treated with OAC [DOACs: *n* = 234 (75%); VKAs: *n* = 76 (25%)], CMBs were present in 86 (28%) patients; of these, 66 (77%) received DOACs. In both groups, CMBs were associated with an increased risk for the composite outcome: VKAs: HR 3.654 [1.614; 8.277]; *p* = 0.002; DOACs: HR 2.230 [1.233; 4.034]; *p* = 0.008. Patients with CMBs had ~50% higher absolute rates of the composite outcome compared to the overall cohort, with a comparable ratio between treatment groups [VKAs 13/20(65%) vs. DOACs 19/66(29%); *p* < 0.01]. The VKA-group had a 2-fold higher IS [VKAs:4 (20%) vs. DOACs:6 (9%); *p* = 0.35] and a 10-fold higher ICH rate [VKAs: 3 (15%) vs. DOACs: 1 (1.5%); *p* = 0.038]. No significant interaction was observed between type of OAC and presence of CMBs. DOAC-patients showed a significantly better functional outcome (OR 0.40 [0.17; 0.94]; *p* = 0.04).

**Conclusions:**

In AF-stroke patients treated with OAC, the presence of CMBs was associated with an unfavorable composite outcome for both VKAs and DOACs, with a higher risk for recurrent IS than for ICH. Strokes were numerically higher under VKAs and increased in the presence of CMBs.

**Clinical trial registration:**

http://www.clinicaltrials.gov, Unique identifier: NCT03826927.

## Introduction

Due to the more favorable safety profile regarding intracranial hemorrhage (ICH), direct oral anticoagulants (DOACs) and not vitamin K antagonists (VKAs) have emerged as first-line treatment in secondary ischemic stroke (IS) prevention related to non-valvular atrial fibrillation (AF) (1). The prevalence of cerebral microbleeds (CMBs)—small brain hemorrhages associated with cerebral small vessel disease (SVD) upon heme-sensitive magnetic resonance imaging (MRI)—increases with age and in patients with AF (2–4).

The presence of CMBs in stroke patients receiving antithrombotic medication for secondary prevention has been shown to be associated with an increased risk of both ICH and recurrent IS, with the absolute risk being higher for IS (5–9). However, in these studies, patients with different stroke etiologies and thus different antithrombotics for secondary stroke prevention—i.e., antiplatelet therapy or oral anticoagulation (OAC)—were included and data on the impact of CMBs on prognosis in different types of OAC, i.e., DOAC vs. VKA, are limited.

Thus, it remains unclear whether the type of OAC in AF-related stroke has a different impact on the clinical course in patients with CMBs than those without. The aim of our study is to address this issue.

## Methods

We included consecutive patients between April 2013 and August 2016 from our prospective, ongoing registry on Novel Oral Anticoagulants in Ischemic Stroke Patients (NOACISP)-LONGTERM, which was conducted at the Stroke Center of the University Hospital Basel. The registry has been approved by the local ethics committee (BASEC PB_2016_00662).

Methodological details have been previously described (8). In brief, patients were treated with OAC for secondary prevention for IS or TIA related to AF. All patients were included in this study, even if they had, e.g., renal insufficiency, mechanical heart valve or active tumor disease. Of note, the decision to treat with a DOAC or a VKA was made based on clinical grounds. Study procedure included a minimum follow-up of at least 3 months and an available brain MRI from the index event, including susceptibility-weighted imaging (SWI) with whole-brain coverage allowing to assess the presence, number and localization of CMBs. We assessed relevant demographic and clinical measures as well as the type of OAC. Outcome measures were: (i) recurrent IS, (ii) ICH, and (iii) death, analyzed separately and as composite outcome. Furthermore, we assessed functional disability based on the modified Rankin Scale (mRS), an ordinal scale measuring the degree of neurological disability, ranging from 0—“no symptoms” to 6—“death,” 3, 6, 12, and 24 months after the qualifying event.

Baseline demographics and clinical data are presented by type of OAC. Continuous data was presented as mean or median and interquartile ratio (IQR) in case of skewed data. Categorical data was presented as count and frequency. Categorical variables were compared using a Chi-square test, continuous variables using a *t*-test, or in case of skewed data a Mann–Whitney U test. Written informed consent was obtained from all patients. Number of events was summarized by type of OAC and presence of CMBs.

The association of CMBs and OAC type with the composite outcome was analyzed using Cox proportional-hazards models. First, we fit a simple model including presence or absence of CMBs, type of OAC (DOAC or VKA) adjusting for the following risk factors: age, sex, hypertension, diabetes, smoking, alcohol consumption, hyperlipidemia, baseline stroke severity (NIHSS), renal function (glomerular filtration rate), as well as additive antiplatelet therapy.

Second, we refit the model adding an interaction term between CMBs and type of OAC to assess whether the type of OAC modifies the effect of CMBs. A survival curve was estimated non-parametrically for the composite outcome stratified to type of OAC and CMBs. The number of each event separately was summarized by type of OAC and presence of CMBs. The number of events in patients treated with DOACs and VKAs was compared using a Chi-square test with continuity correction.

Finally, we assessed the mRS at 3, 6, 12, and 24 months in a mixed-effects ordered logistic regression model using the same two steps approach as described above, additionally adjusted for the pre-mRS. We also assessed the interaction between the mRS, type of OAC and CMB burden with a mixed-effects ordered logistic regression model. Our analysis was conducted in accordance with the STROBE criteria for observational studies (10).

## Results

Baseline demographics and clinical data are shown in Table 1. Overall, we included 310 patients treated with OAC (aged 78.1 ± 9.2years, 46.1% female), with a median follow-up time of 735.5 days (IQR = [498; 814]). Of these, 234 (75%) were treated with DOACs and 76 (25%) with VKAs. CMBs were present in 86 (28%) patients; of these, 66 (77%) were treated with DOACs and 20 (23%) with VKAs, thus comparable to the overall cohort (Supplementary Figure 1). There were no differences between the two treatment groups regarding baseline characteristics, including demographics and vascular risk factors (Table 1), except for renal function (GFR (mean [SD]): VKA 59.1 ml/min/1.7 m^2^ [± 23.7] vs. DOAC 70.2 ml/min/1.7 m^2^ [± 18.3]; *p* < 0.001). In addition, the CHA_2_DS_2_-VASc-score differed by one point in the overall cohort (VKA 6.0 [5.0, 7.0] vs. DOAC 5.0 [4.0, 6.0]; *p* = 0.003), but was the same between treatment groups with CMBs [VKAs: 6 (4.25–6) vs. DOACs: 6 (5–6.75); *p* = 0.32]. The VKA and DOAC groups did not differ regarding presence or localization of CMBs [DOAC 66 (28%) vs. VKA 20 (26%); *p* = 0.863].

**Table 1:** Patient characteristics by anticoagulation at baseline.

	**VKA** **(*n* = 76)**	**DOAC** **(*n* = 234)**	***p*-Value**
**Demographics**			
Age, years (mean) [SD]	79.4 [9.1]	77.7 [9.2]	0.172
Sex = male, *n* (%)	35 (46.1)	132 (56.4)	0.149
**Medication**			
Only DOACs, *n* (%)	–	216 (92.3)	
DOACs/antiplatelet, *n* (%)	–	18 (7.7)	
Only VKAs, *n* (%)	61 (80.3)	–	
VKAs/antiplatelet, *n* (%)	15 (19.7)	–	
**Vascular risk factors**			
Hypertension, *n* (%)	61 (80.3)	175 (74.8)	0.413
Diabetes, *n* (%)	15 (19.7)	47 (20.1)	1.000
Hyperlipidemia, *n* (%)	31 (40.8)	89 (38.0)	0.770
Kidney function, GFR (CKD-EPI: ml/min/1.7) [SD]	59.1 [23.7]	70.2 [18.3]	<0.001
Smoking, *n* (%)	5 (6.6)	15 (6.4)	0.822
Alcohol consumption, *n* (%)	5 (6.6)	17 (7.3)	0.816
**NIHSS at baseline (median [IQR])**	4.0 [2.0, 7.2]	3.0 [1.0, 7.0]	0.447
**Follow-up time, days (median [IQR])**	740 [645; 821]	716.5 [179; 784]	
**Microbleeds**, ***n*** **(%)**	20 (26.3)	66 (28.2)	0.863
Superficial	13 (17.1)	41 (17.5)	
Deep	13 (17.1)	37 (15.8)	
Cerebellum	8 (10.5)	15 (6.4)	
Brainstem	4 (5.3)	9 (3.8)	

SD, Standardized difference; VKAs, Vitamin K antagonists; DOAC, direct oral anticoagulants; GFR, glomerular infiltration rate.

Within the cohort of 310 patients, recurrent IS (*n* = 21) occurred more frequently than ICH (*n* = 7) during the follow-up period. A comparable ratio was observed within the subgroup of patients with CMBs: 10 recurrent IS vs. 4 ICH. Overall, 48 patients died during the follow-up period; of these, 18 had CMBs.

Overall, the composite outcome occurred half as often in DOACs treated patients compared to those on VKAs [46/234 (20%) vs. 30/76 (39%); *p* < 0.01]. The same significant ratio, just with ~50% higher absolute rates compared to the overall cohort, was observed in the subgroups with CMBs [DOACs: 19/66 (29%) vs. VKAs: 13/20 (65%); *p* < 0.01].

Three patients in the VKA group had a mechanical heart valve as an important concomitant diagnosis; each of them had at least one CMB. One of them had an ischemic stroke.

Patients with CMBs had a more than doubled hazard ratio for the combined outcome measure compared to patients without CMBs in the adjusted time-to-event analysis (HR 2.629 [1.616; 4.277]; *p* < 0.001). Treatment with a DOAC was associated with a decreased hazard for the combined outcome of approximately half compared to a VKA (HR 0.482 [0.290; 0.801]; *p* = 0.005).

**Figure 1:**
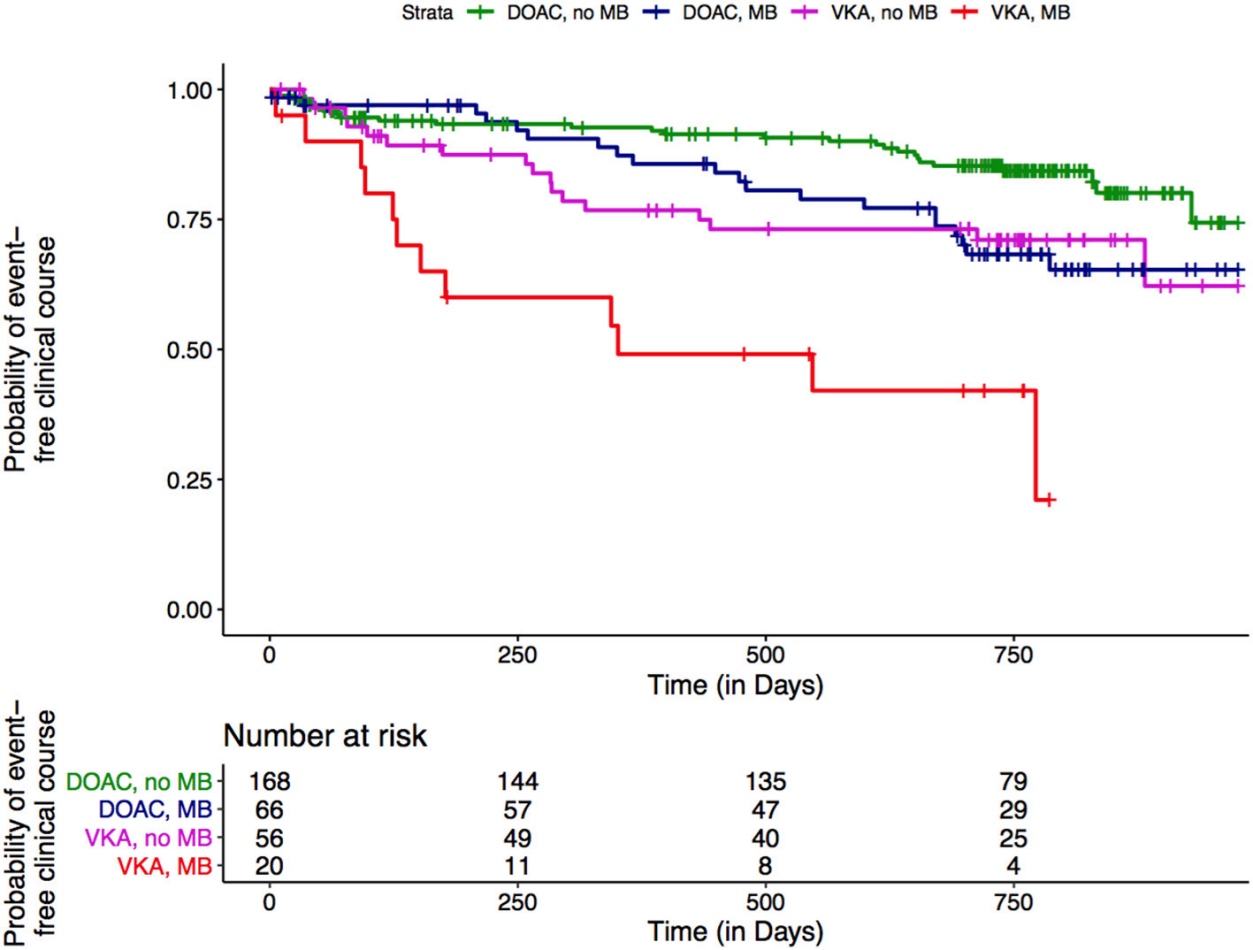
Non-parametric estimation of the survival curves for combined outcome events by type of OAC and CMBs.

In patients treated with VKAs, presence of CMBs was associated with a more than 3-fold hazard increase compared to absence of CMBs in the adjusted analysis (HR 3.654 [1.614; 8.277]; *p* = 0.002; Figure 1, red line vs. purple line). To a lesser extend this was also the case for DOAC treated patients (HR 2.230 [1.233; 4.034]; *p* = 0.008; Figure 1, blue line vs. green line). In line with these findings for both treatment groups, we did not observe a significant interaction between the type of OAC and CMBs in the adjusted analysis, but treatment with DOACs in the presence of CMBs reduced the hazard compared to VKAs by factor 0.610 (HR 0.610 [0.224; 1.660]; *p* = 0.333).

Looking at the outcome events separately, we observed a 2-fold increased rate of recurrent IS in the VKA-group with CMBs compared to the DOAC-group with CMBs [4 (20%) vs. 6 (9%); *p* = 0.35], while the rate of ICH—which was absolutely lower than for recurrent IS—was 10-fold higher in the VKA-group in the presence of CMBs [VKAs: 3 (15%) vs. DOACs: 1 (1.5%); *p* = 0.038].

We did not observe any association of presence or absence of CMBs on functional outcome assessed by mRS, but there was a significantly better functional outcome in patients treated with DOACs (OR 0.40 [0.17; 0.94]; *p* < 0.04) in the adjusted analysis. When including the interaction between the type of OAC and presence of CMBs, no effect modification was found (OR 0.62 [0.10; 3.84]; *p* = 0.61).

## Discussion

In the present study, we observed that the presence of CMBs in patients treated with OAC was associated with a more than 2-fold increased risk for the occurrence of the combined outcome of recurrent IS, ICH or death over a treatment period of 2 years, for both types of OAC, DOACs, and VKAs. Anticoagulation with DOACs was associated with an ~50% reduction of the event rate compared to VKAs. The presence of CMBs increased this risk for both groups, more so in the VKA-group, however, without significant interaction between the type of OAC and the presence of CMBs. Overall, IS emerged more often than ICH, with ICH especially occurring in patients with CMBs treated with VKAs. Finally, treatment with DOACs was associated with a more favorable functional outcome, while the presence of CMBs did not have an impact.

Our study extends previous findings on the impact of the presence of CMBs on outcome in stroke patients on antithrombotic treatment (5–9, 11). While previous studies included various subtypes of strokes treated with different antithrombotics, including OAC but also antiplatelet treatment, we focused on AF-related stroke patients on OAC, looking at the different types of OAC in more detail. Both treatment groups had similar baseline characteristics, except—as expected—regarding renal function.

Recent studies did not report a clear influence of the type of OAC on the risk of ICH in subjects with CMBs (5, 7). In contrast to a meta-analysis of observational studies (9), recent *post hoc* analysis of the RCT NAVIGATE ESUS found no indication of interaction between the effects of rivaroxaban and CMBs for the outcome of ICH (12). Patients in NAVIGATE ESUS had an embolic stroke of unknown primary (ESUS) and were randomized for treatment of aspirin vs. rivaroxaban. The *post hoc* analysis investigated the influence of CMBs. The authors report a 1.5-fold increased risk of recurrent stroke (hazard ratio [HR], 1.5; 95%CI, 1.0–2.3), a 4-fold risk of intracerebral hemorrhage (HR, 4.2; 95%CI, 1.3-13.9), and a 2-fold risk of all-cause mortality (HR, 2.1; 95%CI, 1.1-4.3) in patients with at least one CMB at baseline.

In difference to our study, NAVIGATE ESUS did not differ significantly regarding the risk of recurrent stroke among patients with CMBs (HR, 1.68; 95%CI, 0.79–3.56) and those without CMBs (HR, 0.99; 95% CI, 0.73–1.35). No significant interaction was observed (*P* = 0.33), therefore the authors concluded that CMBs did not appear to influence effects of rivaroxaban on the outcome of ICH.

In our study, besides ICH, we also included recurrent IS and death as outcome measures in our analyses. Of note, we found a comparable association of CMBs for both treatment groups, yet with a higher hazard ratio in the VKA treated patients.

Overall, as illustrated in Figure 1, the clinical course was most unfavorable in subjects with CMBs treated with VKA (*red line*). In contrast, the course of patients without CMBs treated with VKA (Figure 1, *purple line*) was very much comparable to that observed in patients with CMBs on DOACs (Figure 1, *blue line*). Overall—since the effect of CMBs was observed for both types of OAC—there was no clear interaction. Still, Figure 1 illustrates a shift toward a more unfavorable course for both, type of OAC and presence of CMBs, underscoring the potential benefits of DOACs over VKAs in the light of CMBs. In our real-life data, the composite outcome occurred twice as often in the VKA treated group, with an ~50% higher absolute rate in the presence of CMBs. In line with this, CMBs approximately double the risk for an outcome event in the time-to-event analysis, while it was halved in the DOAC- compared to the VKA-group. In the presence of CMBs, the effect of a DOAC treatment was especially observed for ICH, being aware that the numbers of this outcome event were overall low. In the CROMIS-2 study, there also was a lower proportion of DOAC-exposed patients among those who suffered an ICH compared to VKA exposure (14% vs. 86%), however, without reaching statistical significance (5). The MOASIS-AF study investigated 393 AF-patients with an acute IS and present CMBs, of whom 285 were treated with VKAs and 108 with DOACs. A significant association with the composite outcome of stroke, myocardial infarction, and vascular death was observed in the VKA-group (adjusted HR, 2.12; 95% Cl, 1.32–3.43), whereas no association was found in the DOAC-group (adjusted HR, 1.42; 95% Cl, 0.49–4.10) (13).

As in our previous study (8), CMBs were not associated with functional outcome as assessed by the modified Rankin Scale (mRS). In contrast, this was the case with respect to the type of OAC, with a better functional outcome in patients treated with a DOAC. This could be related to the lower rate of IS and ICH in this group.

Our study has some limitations. First, our data are observational rather than randomized. However, our findings are based on real-world data of patients treated at a comprehensive and specialized institution, with a prospective assessment of meaningful clinical endpoints, including cerebrovascular events, mortality, and functional outcome.

Second, we did not perform longitudinal MR-imaging in our cohort, thus we cannot comment on the course of CMBs under different types of OAC. Two observational studies compared the progression of CMBs in IS patients with AF receiving DOACs or VKAs over 12 and 34 months, respectively. Both studies found an increase of CMBs in the VKA compared to the DOAC groups (14, 15). Another retrospective study also provided evidence that the number of CMBs is lower in DOAC than VKA-associated ICH (16). Our treatment groups did not differ regarding presence and localization of CMBs at baseline (8) and our study focussed on the analysis of the prognostic effect of CMBs in a representative cohort of AF-stroke patients treated with different types of OAC.

Third, both treatment groups differed regarding renal function with a lower GFR in the VKA-group. Severe chronic kidney disease (CKD) is a reason to prefer VKAs instead of DOACs, but CKD is also an independent risk factor of both ischemic and hemorrhagic strokes and cerebral small vessel disease (17, 18). Moreover, the CKD stages correlate with the frequency of CMBs (19, 20), possibly explained by common pathogenesis of end-organ damage (21). However, we did not find a difference between the frequency of CMBs between the two treatment groups, and we adjusted for renal function in our analyses.

The CHA_2_DS_2_-VASc-score surprisingly differed between the two treatment groups, although most of their individual variables were very similar (Table 1). Since we did not measure all individual variables, we can only assume that this is a cumulative effect, since all variables in the VKA group were slightly “worse” and thus may have added up to a significant difference. Importantly, the CHA_2_DS_2_-VASc-score was the same between the treatment groups with CMBs. Due to the limited number of outcome events, in particular ICH, we refrained from adjusting for the CHA_2_DS_2_-VASc-score.

We also included patients with mechanical heart valves in our analysis, which is known to be a risk factor for thromboembolic complications (22) and was probably the reason why the physician chose a VKA instead of a DOAC (23, 24). However, there were only 3 patients, one of whom suffered an ischemic stroke. If these patients were excluded, the difference in ischemic strokes would be only slightly decreased, while it would be even greater in the composite outcome and in ICH and deaths. Overall, however, this would not have a major impact on our results, therefore we included these patients in our analyses.

Lastly, the small number of events—especially ICH—prohibits the use of a multistate model. However, from a clinical perspective, the low numbers of outcome events indicate that for both types of OAC, subjects with CMBs were not exposed to exceedingly high risk.

Our study has several strengths. It is a prospective cohort study with a comprehensive and standardized clinical assessment and regular follow-ups over the entire period, thus reflecting current standard of care of AF-stroke patients. Patients were treated and enrolled at our comprehensive stroke center, being the referral center for the entire north-western region in Switzerland. Therefore, our cohort is likely to be a representative stroke cohort related to AF. Treatment decisions were made individually by experienced stroke neurologists at our center according to standard operating procedures, thus reflecting current standard of care of AF-stroke patients. All subjects underwent a standardized stroke MRI with the appropriate sequences, and images were evaluated by three experienced readers blinded to treatment and outcome. High data completeness −98.7% had at least one mRS follow-up—accounting for potential confounders reduced the risk of spurious findings. Besides cerebrovascular events, we included mortality and disability in our analyses, thus extending previous results. Finally, in contrast to the previous studies (5, 7, 11), our cohort included a high rate of subjects treated with DOACs, which nowadays is considered the standard of care for non-valvular AF.

In conclusion, in our cohort of patients with an acute IS, or TIA related to AF, the presence of CMBs was associated with an increased risk of the composite outcome—recurrent IS and ICH and death—for both VKAs and DOACs. Although there was no significant interaction, rates were numerically higher in patients on VKAs for all outcomes—especially for ICH—compared to DOACs. Our findings suggest that among AF-stroke patients, the presence of CMBs may be a clinically meaningful marker for treatment decisions. However, randomized controlled trials are needed to prove this.

## Data availability statement

The original contributions presented in the study are included in the article/Supplementary material, further inquiries can be directed to the corresponding author/s.

## Ethics statement

The studies involving human participants were reviewed and approved by the local Ethics Committee (BASEC PB_2016-00662, former EKBB 52/13; NOACISP-LONGTERM registry). The patients/participants provided their written informed consent to participate in this study.

## Author contributions

BW, NP, and AP contributed to the conception and design of the study. SS performed the statistical analysis. BW wrote the first draft of the manuscript. NP designed the research project and revised the manuscript. All authors contributed to the data collection of the study, commented on previous versions of the manuscript, read, and approved the final version of the manuscript.

## Funding

The authors disclosed receipt of the following financial support for the research, authorship, and/or publication of this article: This study was supported by the Basel Stroke Funds, the Science Funds Rehabilitation Felix-Platter-Hospital Basel and the Neurology Research Pool of the University Hospital Basel. The NOACISP registry was supported by grants from the Science Funds, Bayer AG (Switzerland) and the Stroke Fund of the University Hospital Basel. The funders were not involved in the study design, collection, analysis, interpretation of data, the writing of this article, or the decision to submit it for publication.

## Conflict of interest

DS served on scientific advisory boards for Bayer and Pfizer and received compensation for educational efforts by Stago. CT received travel honoraria from Bayer. SE has received funding for travel or speaker honoraria from Bayer, Boehringer Ingelheim and Daiichi-Sankyo. He has served on scientific advisory boards for Bayer, Boehringer Ingelheim, BMS/Pfizer, and MindMaze and on the editorial board of Stroke. His institutions have received an educational grant from Pfizer, compensation from Stago for educational efforts and research support from Daiichi-Sankyo, the Science Funds [Wissenschaftsfonds] of the University Hospital Basel, the University Basel, from the Wissenschaftsfonds Rehabilitation of the University Hospital for Geriatric Medicine Felix Platter, the Freiwillige Akademische Gesellschaft Basel, the Swiss Heart Foundation, and the Swiss National Science Foundation. NP has served on scientific advisory boards for AstraZeneca, Bayer, Boehringer, BMS/Pfizer, Daiichi-Sankyo and Vifor Pharma. He has received research support from the Swiss Heart Foundation and the Swiss National Science Foundation. The remaining authors declare that the research was conducted in the absence of any commercial or financial relationships that could be construed as a potential conflict of interest.

## Publisher's note

All claims expressed in this article are solely those of the authors and do not necessarily represent those of their affiliated organizations, or those of the publisher, the editors and the reviewers. Any product that may be evaluated in this article, or claim that may be made by its manufacturer, is not guaranteed or endorsed by the publisher.

## Abbreviations

CMBs: Cerebral microbleeds
AF: Atrial fibrillation
OAC: Oral anticoagulant
IS: Ischemic stroke
ICH: Intracranial hemorrhage
mRS: modified Rankin Scale
VKA: Vitamin K antagonist
DOAC: Direct oral anticoagulant
SVD: small vessel disease
MRI: magnetic resonance imaging
SWI: susceptibility-weighted imaging.

## References

[1] Hindricks G Potpara T Dagres N Arbelo E Bax JJ Blomström-Lundqvist C. 2020 ESC Guidelines for the diagnosis management of atrial fibrillation developed in collaboration with the European Association for Cardio-Thoracic Surgery (EACTS) The Task Force for the diagnosis management of atrial fibrillation of the European Society of Cardiology (ESC) Developed with the special contribution of the European Heart Rhythm Association (EHRA) of the ESC. Eur Heart J. (2021) 42:373–498. 10.1093/eurheartj/ehab648 32860505

[2] Poels MMF Vernooij MW Ikram MA Hofman A Krestin GP van der Lugt A & Breteler MMB. Prevalence and risk factors of cerebral microbleeds: an update of the Rotterdam scan study. Stroke. (2010) 41:S103–6. 10.1161/STROKEAHA.110.595181 20876479

[3] Saito T Kawamura Y Tanabe Y Asanome A Takahashi K Sawada J. Cerebral microbleeds and asymptomatic cerebral infarctions in patients with atrial fibrillation. J Stroke Cerebrovasc Dis. (2014) 23:1616–22. 10.1016/j.jstrokecerebrovasdis.2014.01.005 24680089

[4] Sveinbjornsdottir S Sigurdsson S Aspelund T Kjartansson O Eiriksdottir G Valtysdottir B. Cerebral microbleeds in the population based AGES-Reykjavik study: prevalence and location. J Neurol Neurosurg Psychiatry. (2008) 79:1002–6. 10.1136/jnnp.2007.121913 18270235 PMC11090473

[5] Wilson D Ambler G Shakeshaft C Brown MM Charidimou A Al-Shahi Salman R. Cerebral microbleeds and intracranial haemorrhage risk in patients anticoagulated for atrial fibrillation after acute ischaemic stroke or transient ischaemic attack (cromis-2): a multicentre observational cohort study. Lancet Neurol. (2018) 17:539–47. 10.1016/S1474-4422(18)30145-5 29778365 PMC5956310

[6] Wilson D Ambler G Lee KJ Lim JS Shiozawa M Koga M. Cerebral microbleeds and stroke risk after ischaemic stroke or transient ischaemic attack: a pooled analysis of individual patient data from cohort studies. Lancet Neurol. (2019) 18:653–65. 10.1016/S1474-4422(19)30197-8 31130428 PMC6562236

[7] Martí-Fàbregas J Medrano-Martorell S Merino E Prats-Sánchez L Marín R Delgado-Mederos R. MRI predicts intracranial hemorrhage in patients who receive long-term oral anticoagulation. Neurology. (2019) 92:e2432–43. 10.1212/WNL.0000000000007532 31004066 PMC6541433

[8] Hert L Polymeris AA Schaedelin S Lieb J Seiffge DJ Traenka C. Small vessel disease is associated with an unfavourable outcome in stroke patients on oral anticoagulation. Eur Stroke J. (2020) 5:63–72. 10.1177/2396987319888016 32232171 PMC7092732

[9] Wilson D Charidimou A Ambler G Fox ZV Gregoire S Rayson P. Recurrent stroke risk and cerebral microbleed burden in ischemic stroke and TIA: a meta-analysis. Neurology. (2016) 87:1501–10. 10.1212/WNL.0000000000003183 27590288 PMC5075978

[10] Von Elm E Altman DG Egger M Pocock SJ Gøtzsche PC Vandenbroucke JP. The Strengthening the Reporting of Observational Studies in Epidemiology (STROBE) statement: guidelines for reporting observational studies. Ann Intern Med. (2007) 147:573–7. 10.7326/0003-4819-147-8-200710160-00010 17938396

[11] Charidimou A Karayiannis C Song TJ Orken DN Thijs V Lemmens R. Brain microbleeds, anticoagulation, and hemorrhage risk: Meta-analysis in stroke patients with AF. Neurology. (2017) 89:2317–26. 10.1212/WNL.0000000000004704 29117953

[12] Shoamanesh A Hart RG Connolly SJ Kasner SE Smith EE Martí-Fàbregas J. Microbleeds and the effect of anticoagulation in patients with embolic stroke of undetermined source: an exploratory analysis of the navigate ESUS randomized clinical trial. JAMA Neurol. (2021) 78:11–20. 10.1001/jamaneurol.2020.3836 33074284 PMC7573796

[13] Choi KH Kim JH Lee C Kim JM Kang KW Kim JT. Microbleeds and outcome in patients with acute ischemic stroke and atrial fibrillation taking anticoagulants. Stroke. (2020) 51:3514–22. 10.1161/STROKEAHA.120.030300 33028171

[14] Yokoyama M Mizuma A Terao T Tanaka F Nishiyama K Hasegawa Y. Effectiveness of nonvitamin K antagonist oral anticoagulants and warfarin for preventing further cerebral microbleeds in acute ischemic stroke patients with nonvalvular atrial fibrillation and at least one microbleed: CMB-NOW multisite pilot trial. J Stroke Cerebrovasc Dis. (2019) 28:1918–25. 10.1016/j.jstrokecerebrovasdis.2019.03.050 31005561

[15] Umemura T Mashita S Kawamura T. Oral anticoagulant use and the development of new cerebral microbleeds in cardioembolic stroke patients with atrial fibrillation. PloS ONE. (2020) 15:e0238456. 10.1371/journal.pone.0238456 32941455 PMC7498025

[16] Lioutas VA Goyal N Katsanos AH Krogias C Zand R Sharma VK. Microbleed prevalence and burden in anticoagulant-associated intracerebral bleed. Ann Clin Transl Neurol. (2019) 6:1546–51. 10.1002/acn3.50834 31402613 PMC6689674

[17] Toyoda K Ninomiya T. Stroke and cerebrovascular diseases in patients with chronic kidney disease. Lancet Neurol. (2014) 13 :823–33. 10.1016/S1474-4422(14)70026-2 25030514

[18] Lee M Saver JL Chang KH Liao HW Chang SC Ovbiagele B. Low glomerular filtration rate and risk of stroke: meta-analysis. BMJ. (2010) 341:c4249. 10.1136/bmj.c4249 20884696 PMC2948650

[19] Yamashiro K Tanaka R Kurita N Ueno Y Miyamoto N Hira K. Cerebral microbleeds and chronic kidney disease in acute ischemic stroke patients with atrial fibrillation. J Stroke Cerebrovasc Dis. (2020) 29:104650. 10.1016/j.jstrokecerebrovasdis.2020.104650 32024601

[20] Cho AH Lee SB Han SJ Shon YM Yang DW Kim BS. Impaired kidney function and cerebral microbleeds in patients with acute ischemic stroke. Neurology. (2009) 73:1645–8. 10.1212/WNL.0b013e3181c1defa 19917986

[21] O'Rourke MF Safar ME. Relationship between aortic stiffening and microvascular disease in brain and kidney: cause and logic of therapy. Hypertension. (2005) 46:200–4. 10.1161/01.HYP.0000168052.00426.65 15911742

[22] Cannegieter SC Rosendaal FR Briet E. Thromboembolic and bleeding complications in patients with mechanical heart valve prostheses. Circulation. (1994) 89:635–41. 10.1161/01.CIR.89.2.635 8313552

[23] Dangas GD Weitz JI Giustino G Makkar R Mehran R. Prosthetic heart valve thrombosis. J Am Coll Cardiol. (2016) 68:2670–89. 10.1016/j.jacc.2016.09.958 27978952

[24] Eikelboom JW Connolly SJ Brueckmann M Granger CB Kappetein AP Mack MJ. Dabigatran versus warfarin in patients with mechanical heart valves. N Engl J Med. (2013) 369:1206–14. 10.1056/NEJMoa1300615 23991661

