# Supplementary material for: Impact of type of oral anticoagulants in patients with cerebral microbleeds after atrial fibrillation-related ischemic stroke or TIA: Results of the NOACISP-LONGTERM registry
Source: Front Neurol. 2022 Sep 20;13:964723. doi: 10.3389/fneur.2022.964723 (PMC9531011; doi:10.3389/fneur.2022.964723)
Supplement: Supplementary file 1 [file Presentation_1.pptx]

## Slide 1
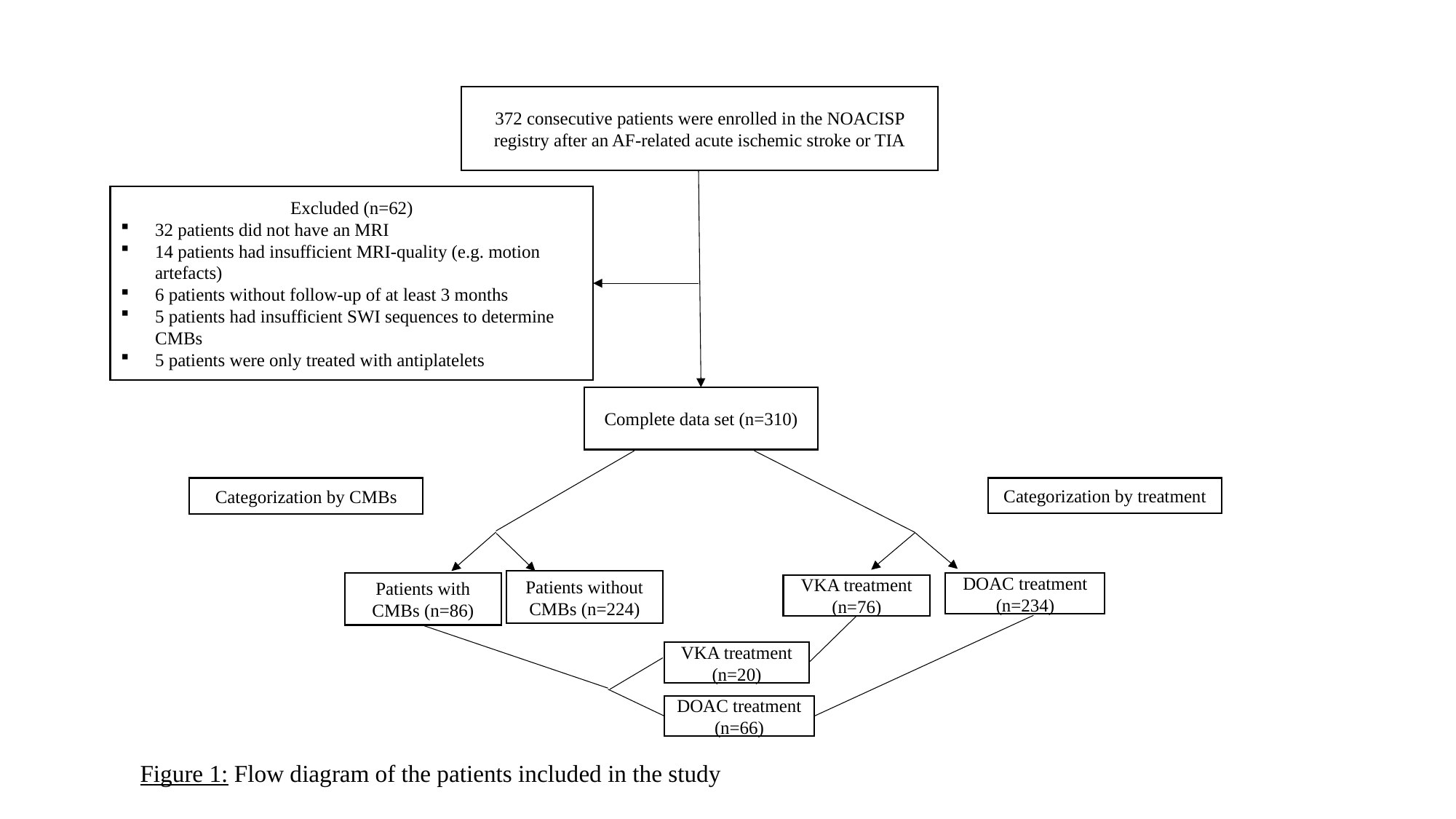

372 consecutive patients were enrolled in the NOACISP registry after an AF-related acute ischemic stroke or TIA
Excluded (n=62)
32 patients did not have an MRI
14 patients had insufficient MRI-quality (e.g. motion artefacts)
6 patients without follow-up of at least 3 months
5 patients had insufficient SWI sequences to determine CMBs
5 patients were only treated with antiplatelets
Complete data set (n=310)
Categorization by CMBs
Categorization by treatment
Patients without CMBs (n=224)
Patients with CMBs (n=86)
DOAC treatment (n=234)
VKA treatment (n=76)
VKA treatment (n=20)
DOAC treatment (n=66)
Figure 1: Flow diagram of the patients included in the study
